# A physician targeted intervention improves prescribing in chronic heart failure in general medical units

**DOI:** 10.1186/s12913-018-3009-x

**Published:** 2018-03-23

**Authors:** Chong Chyn Chua, Anastasia Hutchinson, Mark Tacey, Sumit Parikh, Wen Kwang Lim, Craig Aboltins

**Affiliations:** 1 Department of Medicine, Northern Health, 185 Cooper Street, Epping, VIC Australia; 2 The Northern Clinical Research Centre, Northern Health, Epping, VIC Australia; 3 School of Nursing & Midwifery, Deakin University, Epping, VIC Australia; 4 Northwest Academic Centre, The University of Melbourne, Melbourne, VIC Australia

**Keywords:** Chronic heart failure, Quality improvement, Health professions education, Audit and feedback, Hospital medicine

## Abstract

**Background:**

Despite strong evidence for beta-blockers and angiotensin-converting enzyme inhibitors (ACEI) or angiotensin receptor blockers (ARB) in chronic heart failure (CHF), they have been under-utilised especially in general medical units. We aim to evaluate the effectiveness and feasibility of a physician-targeted quality improvement intervention with education and feedback on the prescription of beta-blockers and ACEI/ARB for CHF management in an inpatient setting.

**Methods:**

We conducted an interrupted time series study between January 2009 and February 2012. A two-stage intervention was implemented. Between November 2009 and January 2011, a structured physician-oriented education program was undertaken. From February 2011, quarterly performance feedback was provided to each medical unit by a senior clinician. Medical notes of patients admitted with CHF under general medical units before and during the intervention were prospectively audited. Main outcomes were beta-blockers and ACEI/ARB prescription rates, and 180-day readmission rates for CHF.

**Results:**

Four hundred and sixty-eight patients were included in this study. Structured education program was associated with a significant rise in beta-blockers prescription rates from a baseline of 60 to 92% (*p* = 0.003), but a non-sustained rise in ACEI/ARB prescription. Regular performance feedback resulted in a further sustained increase in ACEI/ARB prescription rates from 62 to 93% (*p* = 0.028) and a positive trend for beta-blockers with rates maintained at 89%. There was a reduction in 180-day readmission rates that correlated with the improvements in beta-blocker (*p* = 0.030) and ACEI/ARB (*p* = 0.035) prescription.

**Conclusion:**

Implementation of a structured education program with regular performance feedback was durable and was associated with improvements in appropriate prescribing and an observed decrease in CHF-related readmissions.

## Background

Exacerbation of chronic heart failure (CHF) is a leading cause of hospitalisation worldwide. CHF is estimated to cost more than $1 billion per year in Australia and more than $34 billion per year in the United States of America (USA), with hospitalisation accounting for two-thirds of the total expenditure [1–3]. Prevalence of CHF in the Australian, European and USA population is approximately 6.5% in ages 60 and over, rising exponentially in older age groups [4].

There is compelling evidence that the use of certain beta-blockers and an angiotensin-converting enzyme inhibitor (ACEI) or angiotensin receptor blocker (ARB) can alleviate symptoms, reduce hospitalisations and extend the survival of patients with CHF [5, 6]. International CHF management guidelines strongly recommend using these medications as first-line therapy in patients with CHF [7–9].

Multiple studies have observed underutilisation of these medications in patients hospitalised for exacerbation of CHF, especially among general medical units. Prescription rates for ACEI or ARB have been reported to be between 58.7 to 89.2%, and beta-blockers between 10.4 and 80.1% for hospitalised patients with CHF [10–14]. In-hospital initiation of medical therapy has been associated with higher continuation rates post discharge [15].

Translating clinical guidelines to clinical practice is challenging. Multiple methods have been used to change physicians’ practices: audit and feedback, reminders, education, local consensus processes, financial incentives and financial penalties [16]. Systematic reviews have reported that multifaceted interventions with a combination of two or more strategies are more consistent in promoting behavioural change among health professionals [17].

This study aims to report the effectiveness of structured education, audit and feedback as a quality improvement initiative targeted at physicians on the prescription of evidence-based medications in CHF management in general medical units of an outer metropolitan teaching hospital.

## Methods

### Study design and setting

This was an interrupted time series study conducted between January 2009 and February 2012 at The Northern Hospital, a 320 bed outer metropolitan public teaching hospital catering to a community of approximately 728,000 people in North of Melbourne, Victoria, Australia. There were six general medical units during our study period.

### Quality improvement intervention

A two-stage intervention targeting all physicians and junior medical doctors in general medical units was implemented.

#### Stage one

Stage one focused on structured education program and dissemination of CHF management guidelines involving physicians and junior doctors at all levels of training. A hospital-based CHF management guideline was developed with emphasis on two key performance measures: 1) prescribing evidence-based beta-blockers, 2) prescribing ACEI or ARB. A senior physician presented the guideline regularly during medical grand rounds and doctors’ education sessions in the allocated period. Printed materials such as posters and policies were distributed around the hospital.

#### Stage two

Rates of prescription of beta-blockers and ACEI/ARB for patients under each general medical unit was audited and fed back to each unit. Feedback of audit results was provided quarterly to doctors of each general medical unit by a senior physician in both written and verbal format. The audit results were also made available to all medical units, with results presented regularly in the broader general medical unit meetings. The average beta-blocker and ACEI/ARB prescription rates across all medical units were provided for benchmarking.

There was no specific intervention by researchers in the management of individual patients and individual patient level data was not routinely made available to doctors.

### Data collection

Patients were eligible for auditing if they were discharged alive from a general medical unit with a primary diagnosis of heart failure. This was defined as a International classification of diseases 10th Revision (ICD-10) diagnostic-related group (DRG) code of F62A (heart failure with catastrophic complications) or F62B (heart failure without catastrophic complications). The term ‘catastrophic complications’ relate to the amount of resources used during that episode as determined by a nationwide standardised case complexity matrix. The use of ICD-10 codes for case identification was a reliable method as it was compulsory for every patient to have a discharge summary that was coded with ICD-10 codes to ensure appropriate assignment of DRG, which impacted on funding allocation to the hospital. Patients were excluded from the audit if they were under 18 years of age, did not require an inpatient admission, managed by another specialty medical team within the health service or died during that admission. The health service administrative dataset was used prospectively to identify all potential eligible cases. We audited every second eligible patient with a minimum target of five patients per general medical unit per month. If less than five patients were discharged from a general medical unit in a particular month, all eligible patients from that unit were audited.

Researchers reviewed the discharge prescriptions and recorded the use of evidence-based beta-blockers, ACEI or ARB. The beta-blockers deemed appropriate for use were metoprolol succinate, carvedilol and bisoprolol. Patients on nebivolol were not included in this study as the evidence for nebivolol and its routine use was not available at the time of establishing the project protocol and for the first half of the study period. The proportion of patients prescribed each of the medication classes was calculated as the percentage of potential eligible patients for that treatment modality. If patients met exclusion criteria for the use of beta-blockers (defined as medication allergy, asthma and second or third degree heart block) or ACE/ARB (defined as medication allergy, ACEI related cough, acute kidney injury (defined as abrupt decline in renal function with creatinine increasing to more than 1.5× baseline), hyperkalaemia (exceeding 5.5 mmol/L) or bilateral renal artery stenosis), they were excluded from the denominator for their respective treatment modalities. Of note, the presence of an echocardiogram result and documentation of left ventricular dysfunction was not collected to use for feedback in order to avoid pressure on hospital costs and access to this limited resource.

Baseline data on prescription rates was collected from January 2009 to October 2009. Stage one intervention occurred between November 2009 and January 2011. Stage two commenced in February 2011 and continued through to February 2012.

### Outcomes

The primary outcomes for this study were the proportion of patients with a primary diagnosis of CHF who received an evidence-based beta-blocker where there was no contraindication, and the proportion of patients who received an ACEI/ARB where there was no contraindication.

Secondary outcome measure was the 180-day hospital readmission rates for exacerbation of CHF for all patients in the dataset. Outcomes were collected on a monthly basis from January 2009 to February 2012.

### Statistical analysis

The trends of medication prescriptions were analysed using interrupted time series analysis procedures. The possibility of both an instantaneous shift and progressive increasing or decreasing change in the medication prescription rates per month following the introduction of each stage of the interventions were assessed.

Using the Durbin-Watson statistic and by examining auto-correlation it was determined that there was little evidence of auto-correlation in the beta-blocker and ACE/ARB data series. The trends in prescription rates were generally stationary when taking into account shifts after each intervention and seasonal components of the series, thus only Auto-Regressive and Moving Average (ARMA) processes were explored. Statistical analyses were performed with Stata, version 12 (StataCorp, College Station, Tex, USA), with a two-sided *p*-value of less than 0.05 considered to indicate statistical significance.

### Ethics

This project was registered with the Quality and Patient Safety Unit at Northern Health as a quality improvement (audit and feedback) activity and approved for publication by the Northern Health Low-Risk Research Ethics committee.

## Results

Between January 2009 and February 2012, a total of 847 patients were discharged from the general medical units of The Northern Hospital with a primary diagnosis of CHF exacerbation. Of which, we audited the medical records of 468 (55%) patients. The mean age of audited patients was 78 (±10.4) years, with 52% of total patients being female. Baseline characteristics of patients were similar between the audited and non-audited groups (Table 1). During the intervention period the mean quarterly discharge rate for patients with CHF was 88 patients, of these an average of 58 (66%) were included in the audit each quarter.

**Table 1:** Baseline patient characteristics (*N* = 847)

	Audited (*N* = 468)	Not Audited (*N* = 379)
Number of separations	664	560
Age, mean (SD) (years)	77.9 (±10.4)	77.7 (±10.2)
Female (%)	345 (52%)	308 (55%)
Severity of CHF based on DRG code		
F62A	236 (36%)	202 (36%)
F62B	428 (64%)	358 (64%)
Discharge destination		
Private residence	530 (80%)	421 (75%)
Aged care facility	34 (5%)	24 (4%)
Transfer to other healthcare facility	95 (14%)	67 (12%)

*Abbreviations*: *SD* standard deviation, *CHF* chronic heart failure, *DRG* diagnostic-related group, *F62A* heart failure with catastrophic complications, *F62B* heart failure without catastrophic complications

### Primary outcome

**Fig. 1:**
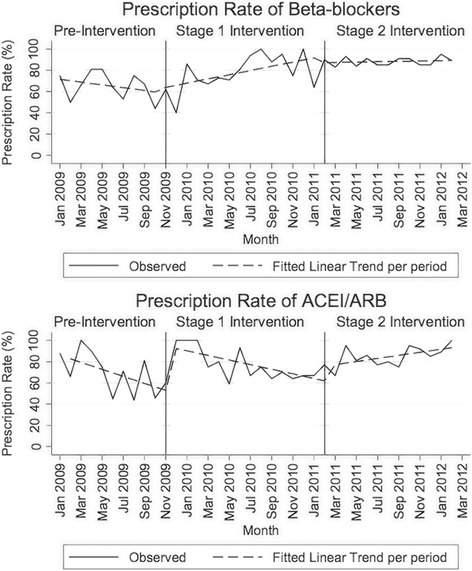
Observed rate and linear trends by intervention period for beta-blockers (top) or ACE/ARB (bottom) prescription

Overall, the prescription rates of both beta-blockers and ACEI/ARB for patients discharged with a primary diagnosis of CHF improved throughout the study. Prescription rates of beta-blockers and ACEI/ARB across the intervention periods can be seen in Fig. 1. A summary of results from time series analysis by intervention period for beta-blockers and ACEI/ARB is shown in Table 2.

**Table 2:** Interrupted time series analysis by intervention period for the prescription rates of beta-blockers and ACEI/ARB

	Beta-blockers			ACEI/ARB		
	Percentage (%)	95% CI (%)	p-value	Percentage (%)	95% CI (%)	*p*-value
Baseline trend						
Prescription rate at month zero	72.8	60.3 to 85.3	< 0.001	89.2	76.7 to 100	< 0.001
Monthly change in prescription rates	−1.3	−3.3 to 0.7	0.198	−3.3	−5.2 to − 1.4	0.001
Stage one intervention						
Initial shift in prescription rates due to intervention (effects lagged by 1 month)	+ 2.2	− 11.3 to 15.8	0.747	+ 41.6	24.4 to 58.6	< 0.001
Subsequent monthly change in prescription rates	+ 3.3	1.1 to 5.4	0.003	+ 1.1	−1.4 to 3.6	0.377
Stage two intervention						
Initial shift in prescription rates due to intervention (effects lagged by 1 month)	−4.6	− 38.9 to 29.6	0.791	+ 13.7	−7.8 to 35.1	0.213
Subsequent monthly change in prescription rates	−1.8	−6.2% to 2.5%	0.415	+ 3.6	0.4% to 6.9%	0.028

*Abbreviations*: *CI* confidence interval, *ACEI* angiotensin-converting enzyme inhibitors, *ARB* angiotensin receptor blocker

The use of beta-blockers rose significantly during stage one of our intervention, with an average increase of 3.3% per month (95%CI: 1.1% to 5.4%, *p* = 0.003) over the 15-month period. The prescription rate for beta-blockers increased from 60% before the intervention, to 92% at the conclusion of stage one. Throughout stage two of our intervention, there was no significant shift (*p* = 0.791) or change to the underlying trend (*p* = 0.415) in the prescription of beta-blockers with the rates stabilising to a range of 87 to 89% over the remainder of the study period.

ACEI/ARB prescription rates had a significant initial rise of 41.6% (95%CI: 24.4% to 58.6%, *p* < 0.001) from 53 to 94.6% at the start of stage one of the intervention, although the impact was lagged by 1 month. Over the remainder of stage one, the prescription rates for ACEI/ARB reduced steadily to a level of 62%. In stage two, we observed a significant sustained upward trend for ACEI/ARB prescription rates at an average rate of 3.6% increase per month (95%CI: 0.4% to 6.9%, *p* = 0.028), also lagged by 1 month, with values reaching 93% by the conclusion of stage two.

### Secondary outcome

**Fig. 2:**
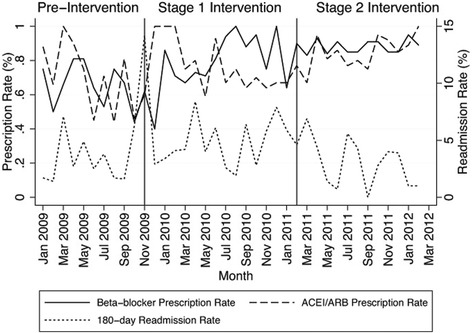
Observed 180 day readmission rates in relation to prescription rates of beta-blockers and ACEI/ARB

As shown in Fig. 2, the average 180-day readmission rate during the pre-intervention period was 3.5% and this had reduced to 3.0% during Stage 2. Time series analysis showed there was a significant negative correlation between 180-day readmission rates for CHF exacerbation and prescription of beta-blocker (*p* = 0.030) and ACEI/ARB (*p* = 0.035), with the 180-day readmission rate for CHF reducing in the month following increases in the prescription rates of beta-blockers and ACEI/ARB.

## Discussion

This study examined the feasibility and durability of implementing a structured education, audit and feedback program on physicians’ prescribing behaviours in the inpatient management of CHF in a real-world public hospital. Our intervention was carried out over a 27-month period to provide a realistic reflection of its applicability. Overall, we have demonstrated that this combination of quality improvement strategies was associated with significant improvements in evidence-based medication use, and may have lead to the observed reduction in the readmission rates for patients hospitalised for exacerbation of CHF.

Studies have evaluated the use of CHF disease management programs or registries to improve the quality of care in CHF patients [18–23]. Most programs adopt a complex multidisciplinary approach involving a team of different health care professionals with various combinations of strategies such as clinical decision support tool-kits, educational sessions, web-based benchmarked quality reports and provision of discharge instructions. The Organized Program to Initiate Lifesaving Treatment in Hospitalized Patients with Heart Failure (OPTIMIZE-HF) registry reported an increase in beta-blocker use from 76.5 to 86.4% and maintenance of ACEI/ARB use at 75.8% in 259 hospitals after a 2-year intervention period [22]. A lesser degree of improvement was noted in an Australian CHF quality improvement program where nine hospitals employed strategies that mainly included a combination of clinical decision support tools and education [24]. Beta-blockers prescription rates on discharge rose from 34 to 52%, and ACEI/ARB rates stabilized at 68% with no significant changes [24]. Although physician education and feedback have constituted parts of these previously published interventions, it has been difficult to evaluate the relative impact of these interventions on physician prescribing behaviour in CHF management.

We observed that our first intervention with a structured education program resulted in significant increases in prescriptions for beta-blockers and ACEI/ARB that was sustained for beta-blockers but not ACEI/ARB. The reason for this discrepancy is not immediately clear, but possible explanations include concerns with medication side effects such as hypotension or renal impairment. The baseline ACEI/ARB prescription rates were also observed to be higher than beta-blockers, which may have accounted for the reduced impact of our first intervention It may also reflect the inconsistent performance of education and guideline dissemination as an intervention in altering physician behaviour as shown in previous studies [25–27]. Nevertheless, education sessions and printed educational materials are often incorporated as part of a more comprehensive approach in view of the feasibility and relative low costs.

In theory, physicians perform better when their performance is monitored regularly and feedback provided with minimal delay to allow appropriate modification of clinical practice [28]. This effect is further enhanced when using achievable benchmarks by comparing their results with their peers [29]. The findings in our study, where audit and feedback of up-to-date information was associated with a notable increase in evidence-based practice, confirm those of a recent Cochrane review that evaluated the impact of audit and feedback in the management of various conditions, not limited to CHF [30, 31]. They concluded that audit and feedback could lead to important improvements in clinical practice especially in physician prescribing [30]. One of our main challenges included the frequent turnover of junior medical staff in the institution i.e. short rotations through general medicine. As such, the delivery of regular and frequent feedback by senior medical staff likely contributed to its effectiveness as an intervention for improving prescription rates [31]. We also hypothesise that the low baseline guideline adherence in our study potentially contributed to the greater effect size observed in our post-intervention prescription rates [30].

Following the implementation of our quality improvement intervention, particularly in Stage 2, there is a notable reduction in the 180-day readmission rates for CHF at our hospital that coincided with the increased prescription of ACEI/ARB and beta-blockers. This is in line with existing strong evidence demonstrating improvements in this outcome [23, 32]. This observed outcome is important as reduced readmission rates have a great implication on the associated healthcare costs and would justify this relatively low cost intervention. In addition, the use of ACEI/ARB and beta-blockers has been shown to reduce mortality in patients with CHF [5, 6]. However, this association was not observed as this study is underpowered to detect changes in this outcome.

In order to reduce the impact on limited resources, we did not mandate the demonstration of left ventricular dysfunction of patients for entry in to this study by echocardiography or other methods. This is important as the evidence of benefit from the assessed medications for patients with heart failure with preserved ejection fraction (HFPEF) is less clear [33, 34]. Patients with HFPEF may have accounted for up to 50% of our cohort and hence would have potentially resulted in an underestimation of the observed reduction in readmission rates. However, this would not have changed the main aim of this study of linking education and feedback to prescribing behaviours.

This study was conducted in a single hospital and the impact of our intervention may not be generalised to other hospitals due to the difference in processes of care and organisational factors. We did not have a control group, thus the improvements in care observed may reflect secular trends in care rather than direct effects of our intervention. A control group was not established as the Australian hospital system was such that junior doctors frequently rotate through different general medical units within the same hospital and may confound the results. Nonethless, utilising interrupted time-series analysis as a statistical technique overcomes many of the limitations of the lack of control group if using historical control. Moreover, to our knowledge, there was no concurrent local or regional intervention implemented for CHF management during the study period to account for our findings. The use of interrupted time-series analysis also allowed us to adjust for random and seasonal variations in medication prescription rates, and confirm that the demonstrated improvements were not merely seasonal fluctuations. Although there is potential for sample bias, inclusion of approximately 68% of discharges each quarter over a period of 3 years and the conservative statistical analysis techniques used should be sufficient to overcome the issue of random sampling error.

General medicine has traditionally had few clinical indicators to support quality practice. The prescription rates of beta-blockers and ACEI/ARB for patients admitted with CHF is a prime candidate given CHF is a common cause of hospitalisation, has high mortality and morbidity, with clear evidence for improved outcomes with these medications. The Australian Council on Healthcare Standards (ACHS) has recently recommended this indicator as a potential measure of the quality use of medications, however widespread reporting has been limited and it is unclear to what extent it is being monitored within individual health services [35]. The findings of our study would support progressing the use of this indicator within hospitals and give evidence for how it might be translated into improved practice.

## Conclusions

In this study, implementation of a structured education program and regular performance feedback was feasible, and associated with durable improvements in appropriate prescribing for CHF with a corresponding decrease in CHF related readmissions. These findings support the more widespread use of these interventions to improve the management of patients admitted with CHF.

## Abbreviations

ACEI: Angiotensin-converting enzyme inhibitors
ARB: Angiotensin receptor blocker
CHF: Chronic heart failure
